# Effects of different embryonic development days on clinical outcomes of freeze-thawed embryo transfer

**DOI:** 10.12669/pjms.41.4.9634

**Published:** 2025-04

**Authors:** Nana Meng, Pengtao Li, Ting Liu, Jiawei Zhai, Meng-yuan Guo

**Affiliations:** 1 Nana Meng, Department of Reproductive Medicine, Baoding Maternal and Child Health Hospital, Baoding 071000, Hebei, China; 2 Pengtao Li, Department of Reproductive Medicine, Baoding Maternal and Child Health Hospital, Baoding 071000, Hebei, China; 3 Ting Liu, Department of Reproductive Medicine, Baoding Maternal and Child Health Hospital, Baoding 071000, Hebei, China; 4 Jiawei Zhai, Department of Reproductive Medicine, Baoding Maternal and Child Health Hospital, Baoding 071000, Hebei, China; 5 Meng-yuan Guo, Department of Reproductive Medicine, Baoding Maternal and Child Health Hospital, Baoding 071000, Hebei, China

**Keywords:** Embryo transfer strategy, Clinical pregnancy rate, Implantation rate

## Abstract

**Objective:**

To compare the clinical outcome of freeze-thawed embryo transfer at different embryonic development days in whole embryo freezing patients of different ages.

**Methods:**

This was a retrospective study. Collected clinical data of 558 patients who underwent freeze-thawed embryo transfer at Reproductive Center of Baoding Maternal and Child Health Hospital from March 2019 to January 12, 2022. The enrolled patients were divided into two groups of 21~35 years old group(n=421) and 35~50 years old group(n=137) according to their ages. Then, three subgroups were established according to the development days of the freeze-thawed embryos under different age groups, D3 subgroup, D5 subgroup and D6 subgroup.

**Results:**

There were no significant differences in biochemical pregnancy rate, clinical pregnancy rate, multiple pregnancy rate, abortion rate, among the three groups in the 21~35 years old group. While significant difference was observed the highest embryo implantation rate in D5 subgroup(*P*<0.05). Meanwhile, in 35~50 years old group, the biochemical pregnancy rate in D5 subgroup was significantly higher than that in D3 and D6 subgroups(*P*<0.05). The clinical pregnancy rate of D5 subgroup was significantly higher than that of D6 subgroup(*P*<0.05), and the clinical pregnancy rate of D5 subgroup was higher than that of D3 subgroup(*P*>0.05). The embryo implantation rate in D5 subgroup was significantly higher than that in D3 and D6 subgroups(*P*<0.05).

**Conclusion:**

Compared with D3 and D6 transfer strategies, D5-blastocyst transfer exhibits more satisfactory clinical outcomes, with the highest clinical pregnancy and embryo implantation rates.

## INTRODUCTION

At presents, a growing number of people have benefited from the improvement of embryo culture conditions, and the emergence of vitrification as a revolutionary ultra-low temperature-storage technique. The development of embryo freezing technology lays a foundation for frozen-thawed embryo transfer, an indispensable component of assisted reproductive technology that is critical in improving women’s live birth rate, pregnancy rate, etc.^1^,^2^ Later, the proposal of the strategy of “whole embryo freezing” sheds new light to patients who are unable to undergo fresh cycle embryo transfer due to various reasons.^3^,^4^ Additional studies also reported that with culture extended to the blastocyst stage, different blastocyst qualities produced no significant impact on neonatal birth outcome.^5^ In addition, the consequences of pregnancy in fresh embryo transfer at the blastocyst stage were not superior to embryo transfer at different stages of the cleavage process.^6^ However, so far, there has been no study comparing the clinical outcomes of day three cleavage-stage embryo transfer, as well as day five and day six blastocyst transfer for patients with different ages. Significantly, the age of women is considered to be the most important factor affecting fertility potential, which significantly decreases after the age of 35.^7^,^8^ Therefore, the present study was carried out to clarify clinical pregnancy outcomes of freeze-thawed D3, D5, and D6 transfer in women aged 21~35 and 35~50 years old, with a purpose to provide reference for developing optimal freeze-thawed embryo transfer strategy for different age groups of patients.

## METHODS

A retrospective analysis was carried out by collecting the clinical data of 558 patients who underwent freeze-thawed embryo transfer in the Reproductive Center of Baoding Maternal and Child Health Hospital from March 2019 to January 12, 2022. The enrolled patients were divided into two groups of 21~35 years old group (n=421) and 35~50 years old group (n=137) according to their ages. Then, three subgroups were established according to the development days of the freeze-thawed embryos under different age groups, i.e., day three cleavage-stage embryo transfer subgroup (D3 subgroup), day five blastocyst transfer subgroup (D5 subgroup) and day six blastocyst transfer subgroup (D6 subgroup).

### Ethical approval:

The study was approved by the Institutional Ethics Committee of Baoding Maternal and Child Health Hospital (No.:2023-01-K005; date: May 12, 2023), and the study met the requirements of the Declaration of Helsinki, and informed consent was provided by participants.

### Inclusion criteria:

- Subjects who underwent whole embryo freezing due to various reasons during the fresh cycle.
- Females with AMH ≥1.1ng/mL.

### Exclusion criteria:

- Patients with habitual miscarriage or repeated implant failure.
- Patients with concomitant adenomyosis and endometriosis.
- Patients with uterine malformations.
- Spouses with history of genetic diseases.

### Embryo scoring:

The quality of cleavage-stage embryos was evaluated in our Center according to the Istanbul Consensus’s cleavage-stage embryo grading scheme. Our Center performed blastocyst culture on the remaining embryos after cleavage-stage embryo freezing. The developed blastocyst was graded based on Garnder blastocyst grading. Embryo freezing was performed on blastocysts of ≥3 stages not simultaneously containing C-grade blastocysts in inner cell mass and trophoblast score.

### Vitrification of embryos:

Vitrification of embryos was performed in strict accordance with the instruction of Vitrification Cooling kit (Kato, Japan). The embryos were transferred to equilibration solution for 9-10 minutes. Then, the embryos were exposed to the vitrification solution for 35-50 s. Embryos were loaded onto the Cryotop (Kitazato, Japan) with a very small volume of vitrification solution, after which they were immediately immersed in liquid nitrogen as quickly as possible.

Thawing of embryos: Embryo resuscitation was performed using the Vitrification Warming kit (Kato, Japan). For resuscitation, the Cryotop was removed from liquid nitrogen and inserted into thawing solution (TS) for one minute and dilution solution(DS) for three minutes according to the instructions of the warming kit, then washed twice for five minutes each in Washing Solution 1 (WS1) and Washing Solution 2 (WS2).After thawing, the embryos were manifested in all blastomeres intact, partial blastomeres intact, or all blastomeres damaged and dead. In general, embryos available for transfer referred to those with the count of blastomeres in the resuscitated embryos after thawing ≥50% of the total cell count. They were transferred to G2 blastocyst culture medium (Vitrolife, Gothenburg, Sweden) and incubated with 5% CO2 at 37°C for further transfer. Routine luteal phase support was given to the patients after embryo transfer.

### Criteria for determining pregnancy outcome:

Blood β-HCG was measured 12-14 days after embryo transfer, and biochemical pregnancy would be confirmed if blood β-HCG was detected to be ≥25 U/L. Transvaginal ultrasound was performed on the 28th to 32nd day after embryo transfer. Clinical pregnancy was confirmed in the presence of gestational sac echo in the uterus and fetal heart beat.

### Statistical indicators:

Biochemical pregnancy rate=number of biochemical pregnancy cycles/total number of transferred embryos×100%; Clinical pregnancy rate=number of clinical pregnancy cycles/total number of transferred embryos×100%; Embryo implantation rate=number of gestational sacs/total number of transferred embryos×100% (the number of gestational sacs in single embryo transfer, n=1); Multiple pregnancy rate=number of cycles with fetal hearts ≥2/clinical pregnancy cycles×100%; Abortion rate=number of miscarriage cycles/number of clinical pregnancy cycles×100%.

### Statistical analysis:

Data was processed using SPSS 27.0 statistical software. The measurement data that conformed to the normal distribution were represented by mean ± standard deviation (*χ̅*±*S*), and inter-group comparison employed one-way analysis of variance. Counting data were presented in percentage (%), and inter-group analysis adopted x^2^ test or Fisher’s exact probability test. *P*<0.05 meant that the difference was statistically significant.

## RESULTS

The enrolled patients were divided into two groups of 21~35 years old group and 35~50 years old group according to their ages to compare the general data and embryo thawing status of patients in different age groups. There were no significant differences in the average age, BMI and proportion of primary infertility between different age groups (*P*>0.05). According to pairwise comparison (Table-I) in the 21~35 years old group, there were significant differences in the comparison of the average number of transferred embryos in D3, D5, and D6 subgroups (*P<*0.05), with the highest in D3 subgroup, followed by that in D5 subgroup, and the smallest in D6 subgroup. In the 35~50 years old group, the average number of transferred embryos in D3 subgroup was significantly higher than that in D5 subgroup (*P*<0.05), but showed no significant difference with that in D6 subgroup (*P*>0.05); and there was no significant difference between D5 and D6 subgroups (*P*>0.05).

**Table-I:** Comparison of general data and embryo thawing status among patients in each group [(*χ̅*±*S*), %]

Groups	Cycles	Average age of females	BMI	Proportion of primary infertility	Average number of transferred embryos
21~35 years old					
D3	254	29.53±2.88	23.86±3.92	62.60(159/254)	1.96±0.2^*^
D5	80	29.74±3.07	23.87±3.61	68.75(55/80)	1.83±0.38^#^
D6	87	28.89±3.21	24.29±4.38	68.97(60/87)	1.75±0.44^&^
35~50 years old					
D3	94	38.00±3.10	24.38±3.64	38.30 (36/94)	1.81±0.4^*^
D5	18	37.39±1.69	25.08±3.63	44.44(8/18)	1.67±0.49^#^
D6	25	37.24±1.85	24.43±3.54	52(13/25)	1.8±0.41^*#^

*Note:* In pairwise comparison, the same letter indicates no significant difference (*P*>0.05); while different letters indicate significant difference (*P*<0.05).

There were no significant differences in biochemical pregnancy rate, clinical pregnancy rate, multiple pregnancy rate, abortion rate, among the three subgroups of D3, D5 and D6 in the 21~35 years old group. While significant difference was observed in embryo implantation rate when compared D5 subgroup with D3 and D6 subgroups (*P*<0.05), with the highest rate in D5 subgroup. Compared with D6 subgroup, D3 subgroup showed higher, clinical pregnancy rate, embryo implantation rate, multiple pregnancy rate, but lower abortion rate (Table-II).

**Table-II:** Comparison of pregnancy outcomes of freeze-thawed transferred embryos at different developmental days in subjects at the age of 21~35 years old

Groups	Cycles	Biochemical pregnancy rate (%)	Clinical pregnancy rate (%)	Embryo implantation rate (%)	Multiple pregnancy rate (%)	Abortion rate (%)
21~35 years old						
D3	254	61.42 (156/254)	56.30 (143/254)	39.64 (197/497) ^*^	32.87 (47/143)	11.19 (16/143)
D5	80	73.75 (59/80)	61.25 (49/80)	49.32 (72/146) ^#^	40.82 (20/49)	12.24 (6/49)
D6	87	62.07 (54/87)	48.28 (42/87)	34.87 (53/152) ^*^	21.4 (9/42)	16.67 (7/42)

*Note:* In pairwise comparison, the same letter indicates no significant difference (*P*>0.05); while different letters indicate significant difference (*P*<0.05).

In 35~50 years old group, D5 subgroup had significantly increased biochemical pregnancy rate than that in D3 and D6 subgroups (*P*<0.05), yet without significant difference in the comparison between D3 and D6 subgroups (*P*>0.05). The clinical pregnancy rate in D5 subgroup was significantly higher than that in D6 subgroup (*P*<0.05); and D5 subgroup showed an increase in the rate than that in D3 subgroup, despite no significant difference between the two subgroups (*P*>0.05). D3 subgroup also revealed increased clinical pregnancy rate than that in D6 group, despite no significant difference between the two subgroups. The embryo implantation rate in D5 subgroup was significantly higher than that in D3 and D6 subgroups (*P*<0.05), while there was no significant difference between D3 and D6 subgroups (*P*>0.05), but with an increasing trend in D3 subgroup compared to D6 subgroup. In terms of multiple pregnancy rate, D3 subgroup showed an increase than that in D6 subgroup (*P*>0.05); and D3 subgroup had slightly higher abortion rate than that in D6 subgroup, with no significant difference. In addition, D5 subgroup had both the highest abortion rate, with no significant difference compared to D3 and D6 subgroups (*P*>0.05) (Table-III).

**Table-III:** Comparison of pregnancy outcomes of freeze-thawed transferred embryos at different developmental days in subjects at the age of 35~50 years old.

Groups	Cycles	Biochemical pregnancy rate (%)	Clinical pregnancy rate (%)	Embryo implantation rate (%)	Multiple pregnancy rate (%)	Abortion rate (%)
35~50 years old						
D3	94	39.36 (37/94) ^*^	34.04 (32/94) ^*#^	24.71 (42/170) ^*^	25 (8/32)	15.63 (5/32)
D5	18	72.22 (13/18) ^#^	55.56 (10/18) ^#^	46.67 (14/30) ^#^	30.00 (3/10)	30.00 (3/10)
D6	25	44.00 (11/25) ^*^	28.00 (7/25) ^*^	17.78 (8/45) ^*^	14.29 (1/7)	14.29 (1/7)

*Note:* In pairwise comparison, the same letter indicates no significant difference (*P*>0.05); while different letters indicate significant difference (*P*<0.05).

## DISCUSSION

In the present study, in the 21~35 years old group, the average number of transferred embryos in D3 subgroup was significantly higher than that in D5 and D6 subgroups (*P*<0.05). However, there was no significant difference in clinical pregnancy rates when comparing D3 subgroup with D5 and D6 subgroups (*P*>0.05); while D5 subgroup showed an increasing trend than the other subgroups, Studies have shown that prolongation of embryo culture to blastocyst stage increases the pregnancy rate.^9^ In this study, despite no significant difference in the comparison of the implantation rate between D3 and D6 subgroups (*P*>0.05), an increasing trend was observed in the former group than that in the latter group. At present, there is still controversy over the impact of the number of transferred embryos on clinical pregnancy rates. Prior research reported a positive correlation of the number of transferred embryos with clinical pregnancy rates, and there would be higher pregnancy rate when more embryos were transferred.^10^ While some other researchers believed that there was no significant correlation between the number of transferred embryos and the pregnancy rate, which was consistent with the results of this study.

It was reported that there was no significant difference in the pregnancy rate between cleavage-stage and blastocyst-stage transfer in patients under 35 years old.^12^ In this study, for females aged 35~50 years old, the average number of transferred embryos in D3 subgroup was significantly higher than that in D5 subgroup (*P*<0.05); but the clinical pregnancy rate in the latter group was increased compared to that in the former group, despite no significant difference between the two subgroups (*P*>0.05); and this rate was significantly increased in D5 subgroup than that in D6 subgroup (P<0.05). Compared with D3 and D6 subgroups, D5 subgroup showed significantly increased implantation rate (*P*<0.05), yet without significant difference in the comparison between the former two groups (*P*>0.05). This is similar to previous studies.^13^ Moreover, a slight increase was observed in D3 subgroup than that in D6 subgroup, which may be related to the following reasons. Firstly, there is significant expansion of D6-blastocysts, with a large amount of fluid in the blastocyst cavity, which may cause incomplete dehydration to form large-sized ice crystals inevitably during freezing, inducing damage to the blastocyst eventually. Secondly, the D6-blastocysts have lost their zona pellucida, and the embryos need to be shaken off by external force during thawing as it was stuck to the Cryotop, which may lead to damage to the blastocysts. Thirdly, the quality of ovum is poor in patients aged >35 years old, and prolonged *in vitro* culture to D6 may increase the risk of damage and the rate of aneuploidy in embryos. Collectively, D6-blastocysts may experience a decrease in clinical pregnancy rate and embryo implantation rate after freeze-thawed embryo transfer.

According to previous studies of single blastocyst transfer, the biochemical pregnancy rate, clinical pregnancy rate, of D5 group were significantly higher than those of D6 group, yet without significant difference in abortion rate between the two subgroups.^14^-^16^ Besides, another study reported the clinical outcomes of frozen-thawed D5 and D6-blastocysts undergoing preimplantation genetic testing. It showed that the pregnancy rate of D5-blastocysts was significantly higher, whereas the miscarriage rate of D5-blastocysts was lower, than that of D6-blastocyst tissue biopsy^17^,^18^. In the present study, there was no significant difference in the abortion rate between the D5 and D6 subgroups (*P*>0.05).

In this study, for patients aged 21~35 and 35~50 years old, the embryo implantation rates in D5 subgroup were both significantly higher than those in D6 subgroup (*P*<0.05), suggesting that the developmental potential of freeze-thawed D5-blastocysts was superior to that of D6-blastocysts. With the prolongation of in vitro culture time, blastocysts with the same or similar quality would present with a series of changes, such as increased count, enlarged blastocyst cavity, filling of blastocyst fluid, and acceleration of metabolism, leading to certain damage to the blastocyst during freeze-thaw cycles. Embryo culture to D6 would also prolong the exposure duration of embryos and result in epigenetic changes in the embryo. Additionally, D6-blastocysts would experience significantly higher incidence of abnormal spindle bodies than that of D5-blastocysts, resulting in higher proportion of aneuploid embryos in D6-blastocysts that might induce the risk of miscarriage and hence decreased implantation rate^19^. Another research calculated the clinical outcomes of freeze-thawed blastocysts in patients under 40 years old and proposed that D5-blastocyst transfer may be the optimal option.^20^

### Limitations:

It includes a small number of patients who were included and no long-term follow-up was conducted. Further research is still needed for confirmation based on large-scale studies with increased number of patients in the future. In addition, prolonged follow-up is also essential to identify long-term effects of cleavage-stage and blastocyst transfer, so as to provide more effective evidence-based medical data for clinical practice.

## CONCLUSIONS

For patients of all age groups, freeze-thawed D5-blastocyst transfer after whole embryo freezing can achieve more satisfactory clinical pregnancy, embryo implantation, compared to cleavage-stage D3 embryo and D6-blastocyst transfer. Moreover, freeze-thawed cleavage-stage D3 embryo transfer may result in increased clinical pregnancy, embryo implantation, multiple pregnancy, and decreased abortion rate compared to D6-blastocyst transfer. Therefore, freeze-thawed cleavage-stage D3 embryo transfer may have an advantage over D6-blastocyst transfer.

### Authors’ Contributions:

**NM and PL** carried out the studies, participated in collecting data, and drafted the manuscript, and are responsible and accountable for the accuracy or integrity of the work.

**TL, JZ and MG** performed the statistical analysis and participated in its design, critical analysis.

All authors read and approved the final manuscript.
